# 3D‐printed wound channels

**DOI:** 10.1111/1556-4029.70421

**Published:** 2026-07-26

**Authors:** Fabiano Riva, Daan Wintermans, Arthur Baert, Hervé Daudigny, Dellaram Anissi, Virginie Magnin

**Affiliations:** ^1^ Centre Universitaire Romand de Médecine Légale Lausanne‐Geneva University Hospital of Lausanne Lausanne Switzerland; ^2^ Ecole Des Sciences Criminelles University of Lausanne Lausanne Switzerland

**Keywords:** 3D printing, ballistic soap, computed tomography, temporary cavity, wound ballistics

## Abstract

Although soft tissue simulants are widely used in ballistic research, glycerine soap is the only material that offers a clear view of the temporary cavity of a wound channel without the use of optical apparatus. However, the latter still shows some inconvenience mostly on its weight and its dehydration process when cut, making it challenging to carry and to store. In an attempt to overcome these issues, the authors tried printing the temporary cavity using 3D‐printable materials, with no intention of producing a faithful reproduction suitable for micrometric analysis. Several shots were made with different firearms and ammunition in order to create temporary cavities in glycerine soap blocks manufactured by Mettler (CH, D). The next step in the process involved the conversion of each cavity into a 3D surface derived from computed tomography (CT) scans, in order to be printed using a 3D printer. The results obtained show clear and light representations of temporary cavities that can easily be stored and manipulated by any attendant or student for educational purposes, for instance. The methodology used and the results obtained are intended solely to illustrate temporary cavities for demonstration purposes in teaching or training. Under no circumstances does this technical note seek to present a new technique for the microscopic measurement of temporary wound cavities.

**Highlights**

- Investigates a practical solution for visualizing temporary cavities in soft tissue simulants.
- Use of technologies such as computed tomography (CT) and 3D printing for temporary cavities visualization.
- Lightweight temporary cavity representation for easy transport and storage.

## 1 INTRODUCTION

Wound ballistics is the part of ballistics that focuses on the analysis of injuries caused by projectiles and on their behavior within biological tissues. In order to provide additional information to the shooting scene reconstructions, forensic ballisticians and pathologists often conduct ballistics tests to better understand the damaging effect of a projectile in a specific situation. According to Maiden, the correct interpretation of bullet wound features, when combined with experimental research, has made this field of study an important tool in investigating criminal acts involving firearms [1].

To reproduce the characteristics of a wound caused by a given type of ammunition or projectile, the forensic ballistician often uses synthetic materials that simulate the way biological tissue responds to being penetrated by a projectile. According to Kneubuehl, a simulant is a material that reacts to bullets in a similar manner to tissue in terms of elasticity, energy absorption capacity, strength, and so on. Simulants are used to model the physical ballistic aspects of wound ballistics. The density of the simulant should be similar to that of tissue. The two soft tissue simulants most commonly used in wound ballistics are gelatine (10% at 4°C) and glycerine soap. Both have a density very similar to that of muscle tissue (density = 1.06 g/cm^3^) [2]. While gelatine has the advantage of transparency due to its elasticity, this only allows the temporary cavity to be visualized by means of a high‐speed camera. On the other hand, glycerine soap exhibits plastic behavior when subjected to a high‐velocity impact. This allows the temporary cavity caused by the projectile to be analyzed simply by cutting the soap block along the projectile's path [3]. This, coupled with the fact that it can be stored for a long time, makes soap an ideal material for demonstrating the effects of temporary cavities, even after a long period of time (e.g. for educational purposes). Despite these qualities, a block of soap measuring 25 × 25 × 40 cm, weighs approximately 25 kg, which makes its transportation tedious. Cutting it in half could enable observation of the temporary cavity while reducing its weight. Unfortunately, the dehydration process accelerates after cutting, resulting in changes to the temporary cavity over time.

This technical note investigates thus a 3D printing‐based procedure that can address the issue of soap blocks deteriorating over time by fixing the temporary cavity using lighter and more portable materials. This document aims to propose a simple, accessible technique for creating demonstrative models that illustrate the temporary cavity produced by a projectile in soft tissue. This last point is key because the aim of this study is not to reproduce the cavity with micrometric precision or replicate the complex biomechanical and microscopic processes that occur in living tissue. Instead, the proposed method seeks to generate a simplified macroscopic representation of the temporary cavity that is sufficiently accurate for educational and demonstrative purposes, enabling a clear visualization of projectile dynamics, energy transfer, and the general mechanisms of tissue disruption associated with projectile penetration.

## 2 MATERIALS AND METHODS

In order to reproduce temporary cavity, shooting tests have been performed using ballistic soap. After shooting, the blocks have been scanned with the use of a computed tomography (CT), a tool often used in forensic medicine. Data on the temporary cavity has been extracted from the resulting scans and used to create 3D models with a 3D printer. The different steps starting from the shooting tests until the 3D printing of the temporary cavity are described in the following sections.

### 2.1 Firearms, ammunitions, and soft tissue simulant

The firearms and ammunitions used in this study as well as their nominal characteristics are summarized in Table 1 [4].

**TABLE 1:** Firearms and ammunitions used in the present study.

Firearm	Ammunition	Caliber	Projectile mass (g/g)	Nominal velocity (m/s)	Nominal energy (J)
SIG SG550	RWS – Soft Point	.223 Remington	3.6/55	965	1658
Glock 17	Winchester SXT9	9 mm Luger	9.5/147	305	442
Glock 17	Ruag Action 4	9 mm Luger	6.1/94	420	538

A single shot per ammunition was fired at a glycerine soap block measuring 25 × 25 × 40 cm, which had been obtained from the soap manufacturer Mettler (CH, D). The shooting distance was approximately 2 m.

For clarity, it is important to emphasize that it is not possible to assess the shape and dimensions of the temporary cavity without cutting the block of soap along the lesion channel. In practice, in order to observe the effects of a projectile passing through a block of soap (and thus the temporary cavity), it is necessary to cut the block in half. This results in two halves of the block, each of which represents half of the temporary cavity (in negative). One way to overcome this alteration of the block caused by the cutting process, is to scan it first with, as previously mentioned, a CT scanner, often available at any Institute of Forensic Medicine [5]. Finally, this method provides an accurate representation of the surface of the cavity inside the block by preserving its shape and without any alteration.

### 2.2 Temporary cavity data acquisition

The glycerine soap blocks have been scanned using a GE Healthcare Milwaukee, USA, Discovery HD750 CT scanner. The following acquisition parameters were used for all blocks: *helical mode, tube potential of 120 kVp, tube current 480 mA, slice thickness 1.25 mm*, *interval 0.4*, *standard algorithm* of reconstruction and utilization of *MAR algorithm* for artifact reduction.

#### 2.2.1 Data extraction

Based on the variation in density, the surface model of the entire temporary cavity, as well as that of the projectile (or fragments), has been isolated and extracted in .STL format using the AW Server software for each shot.

#### 2.2.2 Data processing

First, the STL data generated by the CT scan are imported into the MeshLab software (version 2025.07) to remove unconnected 3D structures (#1 in Figure 1) and smooth the data (the result is showed in Figure 2). This final step is particularly important because metallic objects embedded within the soap (i.e., the projectile and its fragments) introduce artifacts into the CT images [6]. These artifacts consequently affect the quality of the 3D model reconstructed from the CT data. The smoothing procedure allows the metallic particles to be resized without eliminating them, thereby reducing acquisition‐related noise while ensuring their incorporation into the final 3D wound profile.

**FIGURE 1:**
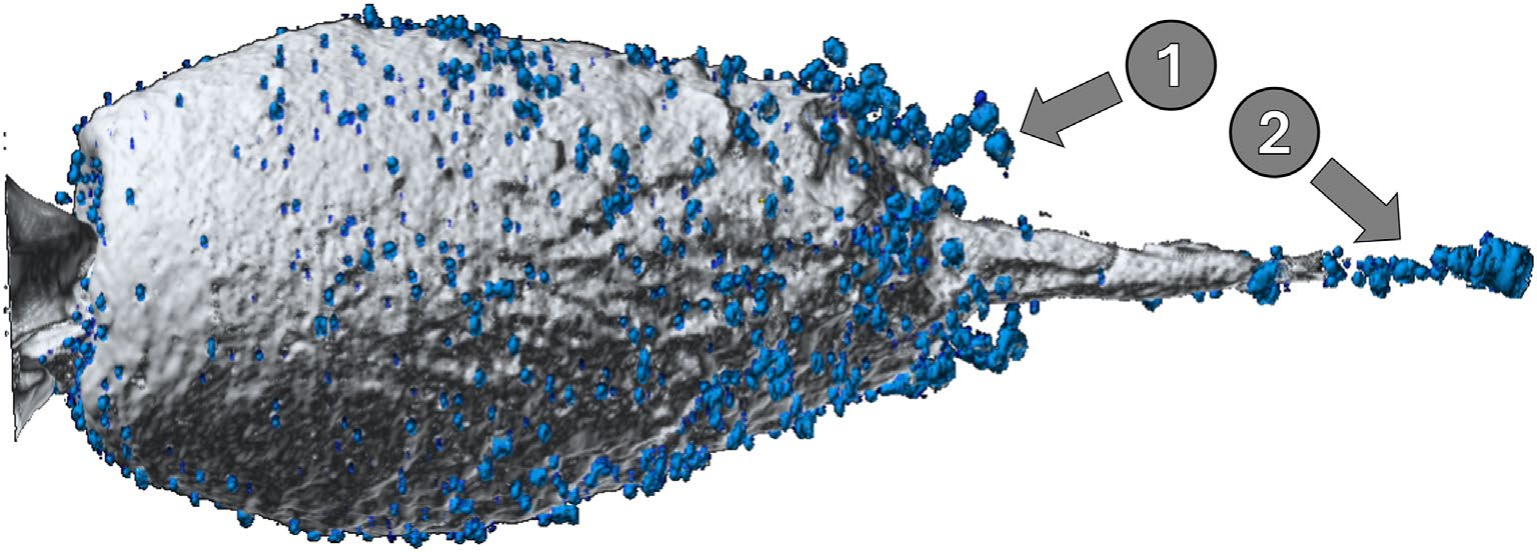
Volumetric data were extracted using the AW Server software. The gray surface corresponds to the temporary cavity in the block of soap, whereas the blue particles illustrate the position of the projectile or its fragments. Preliminary data processing consisted of (1) removing disconnected 3D structures and smoothing the surface, and (2) creating a bridge between the main cavity and the projectile. Hence, the resulting .STL file represents the surface of the temporary cavity. The shooting direction is from left to right.

**FIGURE 2:**
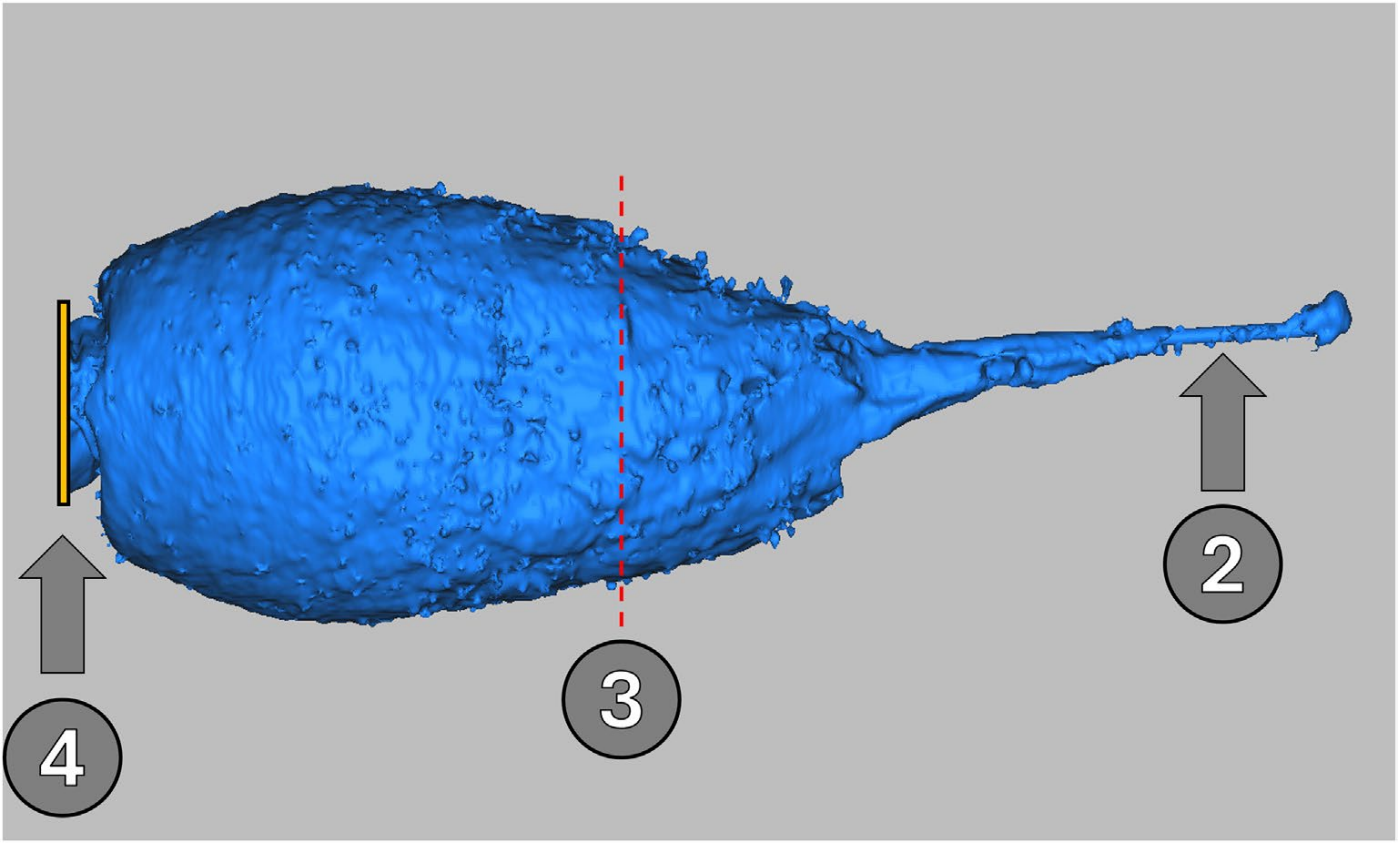
3D representation of the .STL file after preliminary processing. The 3D data are then processed further by cutting the entire structure (red dotted line) and closing the volumes by adding and merging planes with the cavity surfaces (yellow plane). The shooting direction is from left to right.

The processed data are then exported and imported into the CloudCompare software (version 2.12.4) for further processing. The final steps are as follows: (1) connect the main wound cavity to the bullet by adding a basic structure, such as a small cylinder (#2 in Figures 1 and 2); (2) divide the whole structure into two or more pieces, according to the channel length, in order to fit the structure to the boundaries of the 3D printer (#3 in Figure 2); and (3) close the .STL structures by adding and merging a plane to the cavity extremities (#4 in Figure 2). Finally, the resulting 3D structure is exported as two distinct .STL files prior to 3D printing.

### 2.3 3D printing

To print the temporary cavities, a Bambu Lab X1 printer and a PLA (polylactic acid) filament have been used. The G‐code instructions, which establish a link between the .STL files and the 3D printer, have been generated by importing the .STL files into the Bambu Studio V2.3.0.70 slicer and provides the following printing parameters: *layer height* (0.2 mm), *wall loops* (3), *top shell layers* (5), *bottom shell layers* (3), *internal solid infill pattern* (rectilinear), and *sparse infill density* (20%).

The print settings were chosen arbitrarily. Depending on requirements, these settings can be adjusted while still producing sufficiently strong 3D prints. The resulting printing plan is illustrated in Figure 3.

**FIGURE 3:**
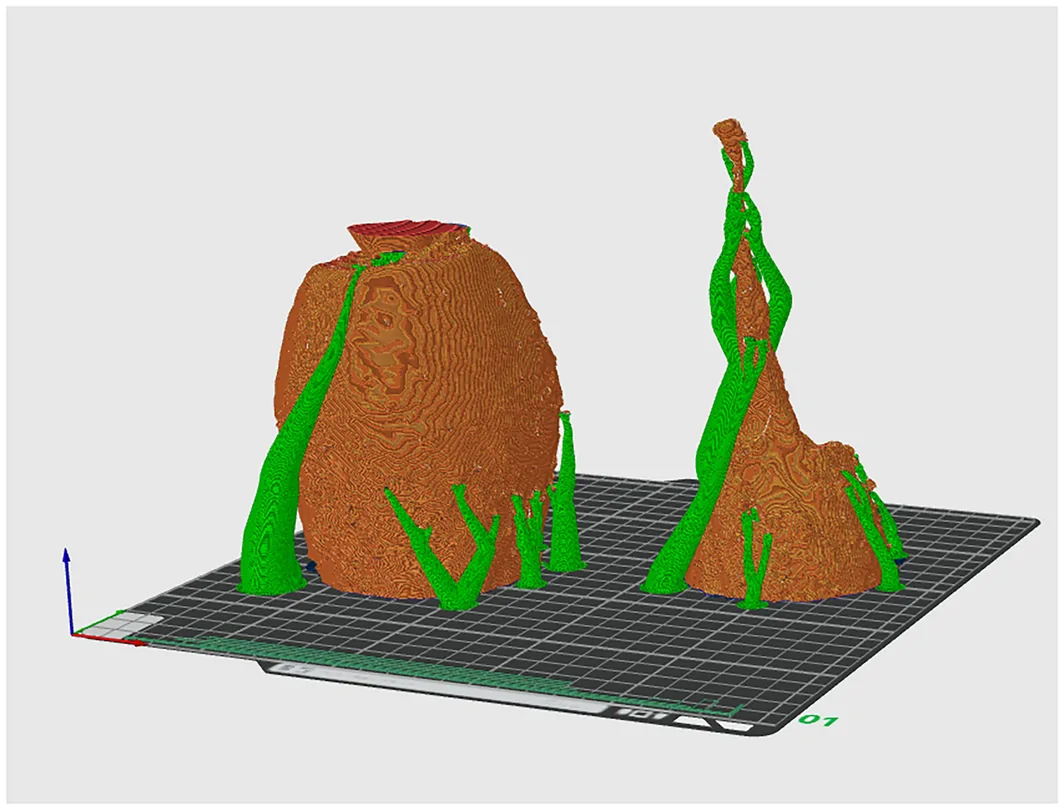
Image showing the printing project in the Bambu Studio slicer. The orange surface represents the object's cavity, whereas the green structure is the support used by the printer to ensure correct printing when the object's slope exceeds 30°.

The printing time for the temporary cavity of the .223 Remington projectile is about 7 h, and the final mass of the model is approximately 283 g. Once printing is complete, the two pieces must be joined together using standard plastic glue (*UHU Hart Kunststoff*), creating the final 3D‐printed temporary cavity with 1:1 dimensions. The following figures show the 3D‐printed models, as well as their respective temporary cavities “frozen” in ballistics soap, for all the projectiles listed in Table 1 (Figures 4 and 5 for the .223 Remington projectile—Figures 6 and 7 and for both 9 mm Luger projectiles).

**FIGURE 4:**
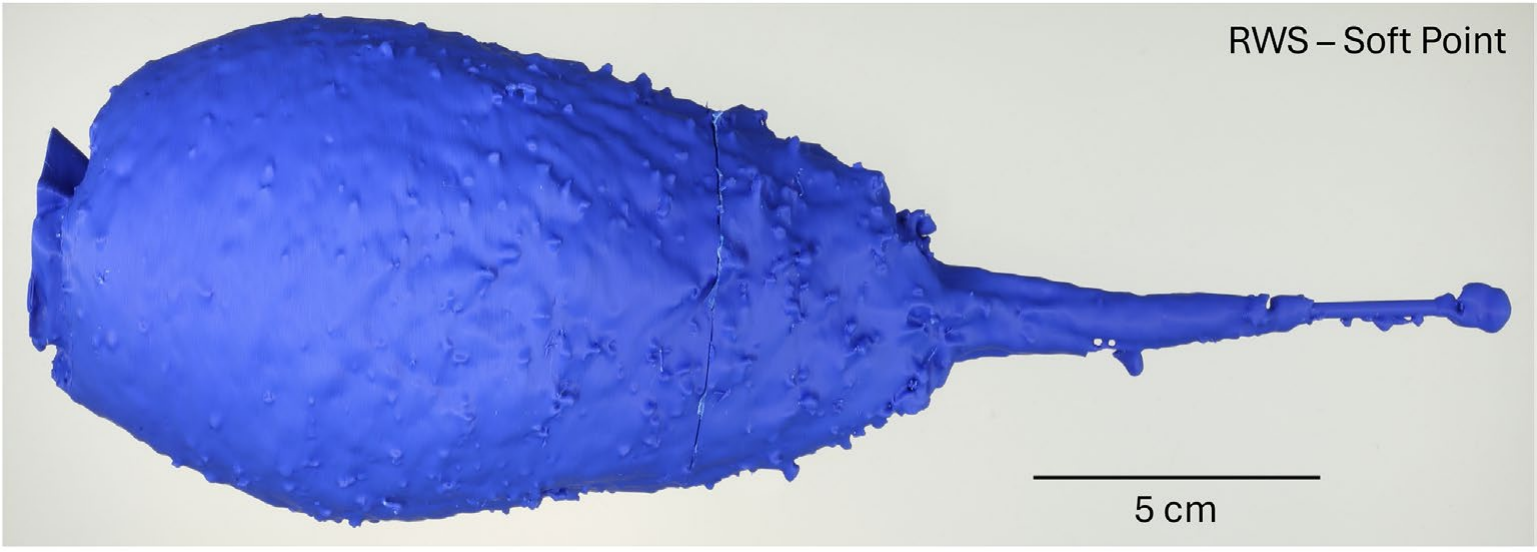
Image of the 3D printed temporary cavity of the .223 Remington Soft Point projectile. The shooting direction is from left to right.

**FIGURE 5:**
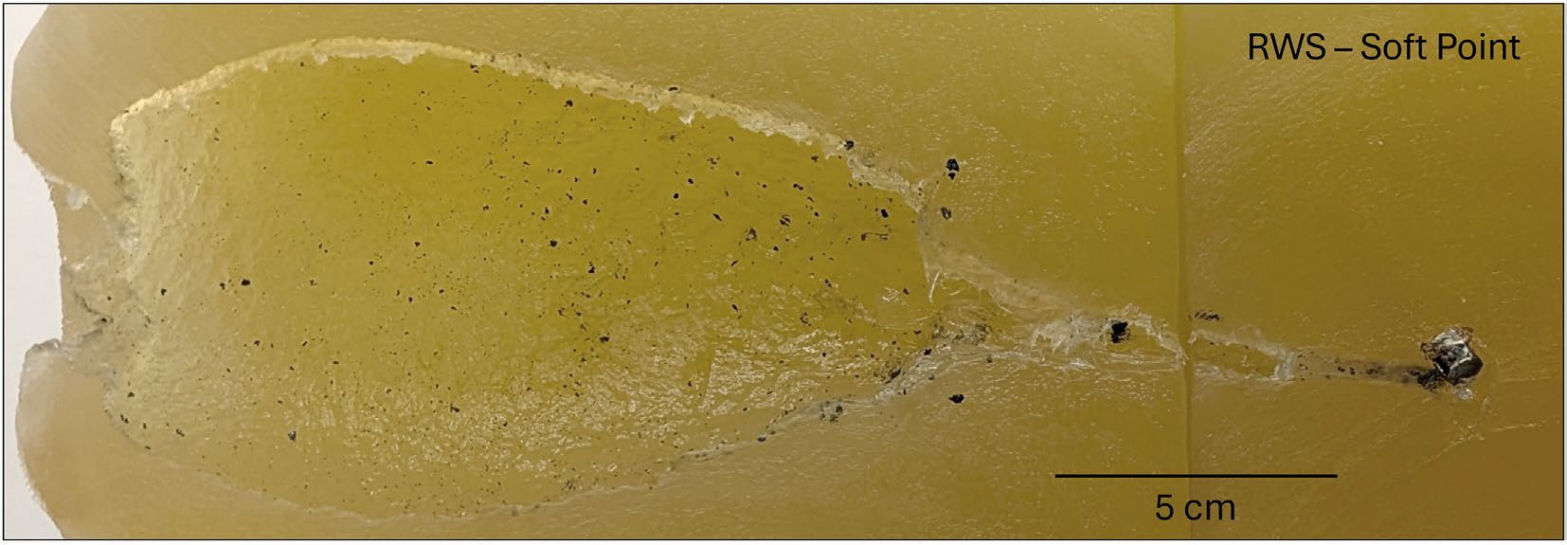
Image showing half of the temporary cavity of the .223 Remington Soft Point projectile in the glycerine soap block, after it was cut along the bullet's path. The shooting direction is from left to right.

**FIGURE 6:**
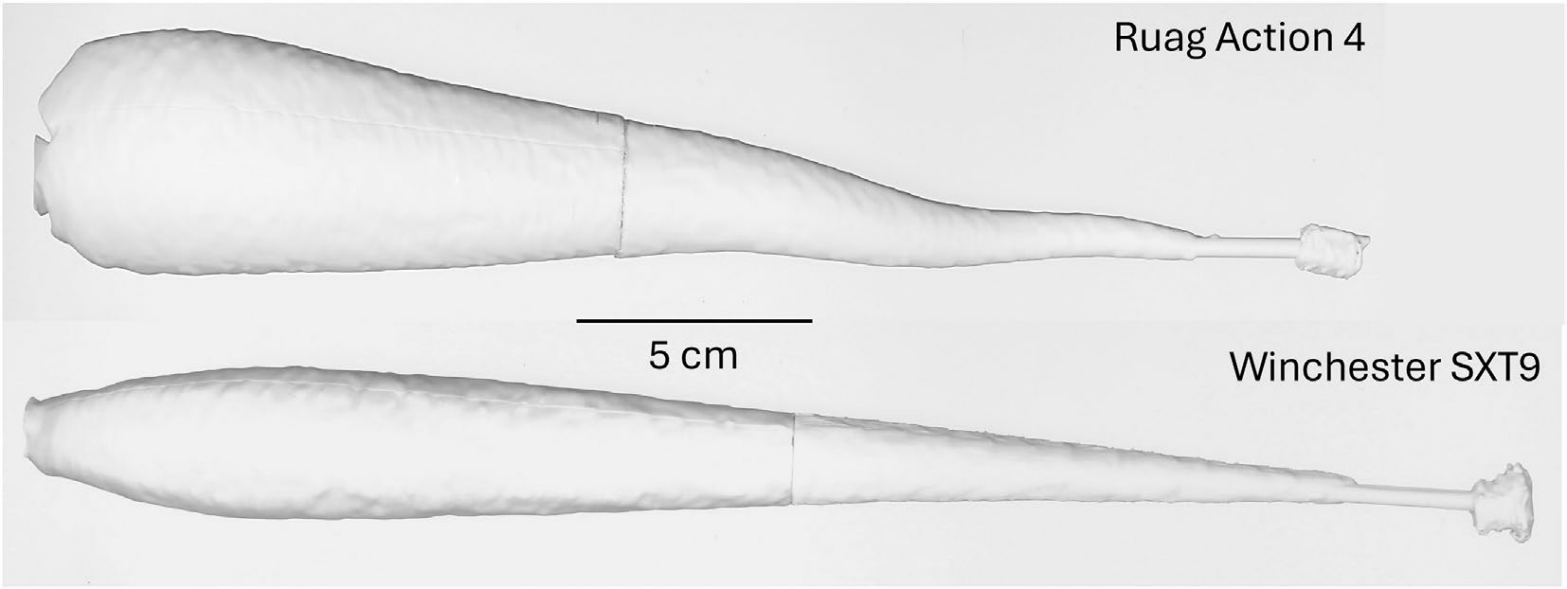
Image of the 3D printed temporary cavity of both 9 mm Luger projectiles (Ruag Action 4 above and Winchester SXT9 below). The shooting direction is from left to right.

**FIGURE 7:**
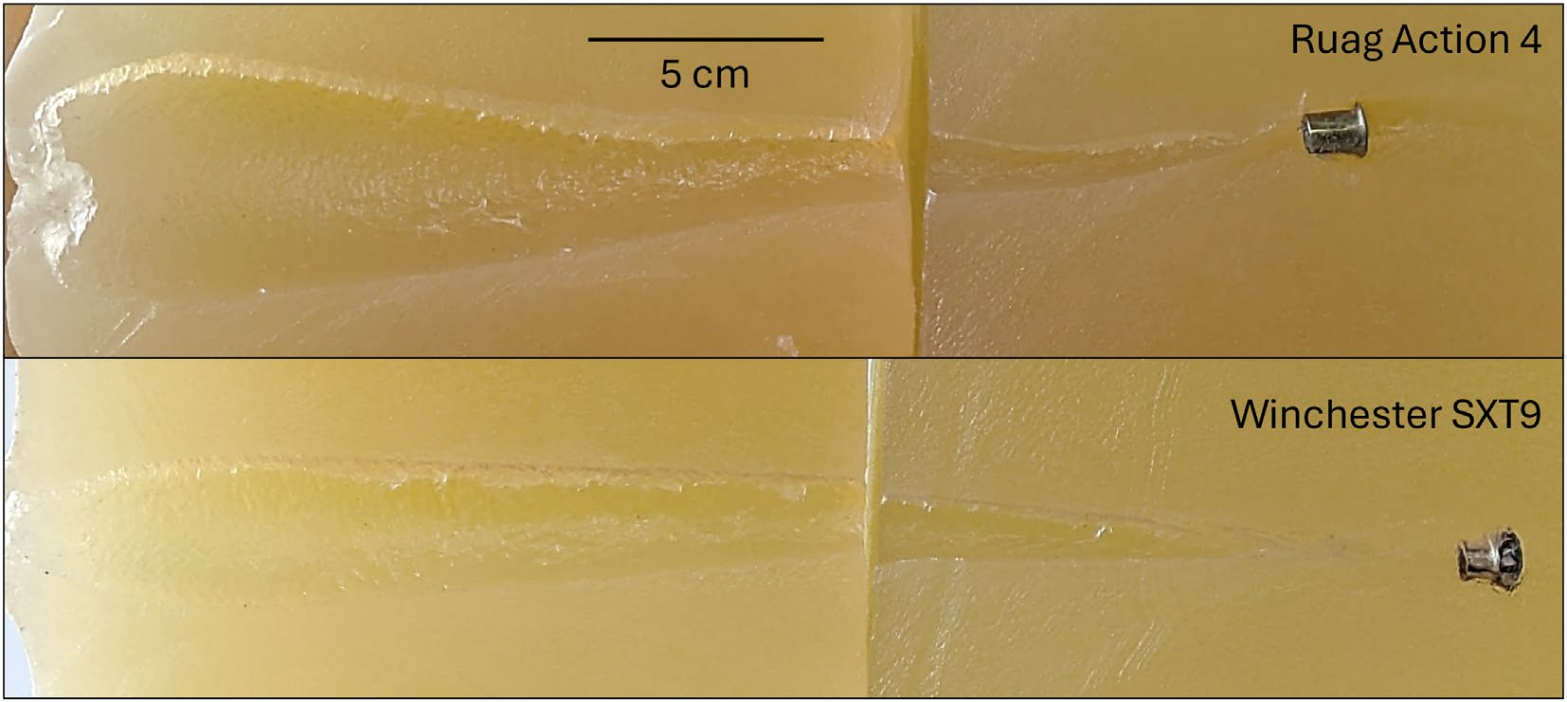
Image showing half of the temporary cavities of both 9 mm Luger projectiles (Ruag Action 4 above and Winchester SXT9 below) in the glycerine soap block, after it was cut along the bullet's path. The shooting direction is from left to right.

Although this was beyond the scope of this study, the main dimensions of the Winchester SXT9 wound cavity (penetration length and maximum width) were measured using the image of the wound channel in the soap (Figure 7 below), the .STL file, and the 3D‐printed model (Figure 6 below). A comparison of these measurements shows no significant difference in the maximum width of the cavity, as well as negligible differences in penetration length (−1.2%) between the image and the printed model. These differences are likely to have been caused by deformations resulting from the cutting of the soap block after scanning. Indeed, when the images were taken, the two halves of the block were no longer a perfect fit, leaving a small gap (Figure 7 below). This consequently resulted in a slight increase in the length measured by the image analysis.

## 3 DISCUSSION AND CONCLUSION

Synthetic materials such as ballistic gelatine or glycerine soap are widely used in forensic ballistics to reproduce and analyze wound patterns produced by different types of ammunition. Each material presents specific advantages and limitations. Although gelatine provides transparency, which makes it possible to observe the projectile trajectory, its elastic properties prevent the temporary cavity from being permanently preserved. This can only be visualized using high‐speed imaging techniques.

By contrast, glycerine soap enables the temporary cavity formed when the projectile penetrates the block to be preserved permanently. This makes it possible to analyze the cavity by physically cutting the material into sections along the bullet's path. However, the use of ballistic soap also presents several practical limitations. Blocks of soap large enough to capture the full cavity structure are heavy and difficult to handle, typically weighing around 25 kg for a block measuring 25 × 25 × 40 cm. Furthermore, once the block is cut open to reveal the cavity, the material begins to dehydrate, which progressively alters the cavity's morphology over time. This degradation can make long‐term storage, transportation, and repeated demonstrations of the cavity structure more difficult.

This technical note presents a method that offers a practical solution to these limitations by combining CT imaging and additive manufacturing. Scanning the interior of the soap block before cutting it and generating a three‐dimensional digital model of the cavity makes it possible to reproduce the structure using lightweight materials through 3D printing. This approach allows portable, durable replicas to be created that can be handled without risking degradation of the original soap block.

Although the temporary cavity's overall dimensions and general morphology are reproduced accurately, minor alterations to its surface details may occur at different stages of the workflow, including CT acquisition, digital segmentation and 3D printing. Nevertheless, these variations do not significantly impact the interpretative value of the model. When it comes to demonstrative purposes, such as educational displays, expert explanations or courtroom presentations, the faithful reproduction of the cavity's overall geometry is far more important than preserving microscopic surface features.

In conclusion, this study demonstrates the feasibility of producing a positive, three‐dimensional replica of a temporary cavity that was originally cast in negative form within ballistic soap. The proposed workflow offers an effective and straightforward method of preserving and reproducing ballistic cavity structures, overcoming the logistical challenges posed by large soap blocks. These replicas could be valuable tools for demonstrating concepts in forensic education, scientific communication, and judicial contexts.

## CONFLICT OF INTEREST STATEMENT

The authors whose names are listed immediately below certify that they have NO affiliations with or involvement in any organization or entity with any financial interest (such as honoraria; educational grants; participation in speakers' bureaus; membership, employment, consultancies, stock ownership, or other equity interest; and expert testimony or patent‐licensing arrangements), or non‐financial interest (such as personal or professional relationships, affiliations, knowledge or beliefs) in the subject matter or materials discussed in this manuscript.

## Data Availability

All data are available from the corresponding author upon reasonable request.
